# Impaction from Adhesion of the Small Colon

**Published:** 1899-06

**Authors:** 


IMPACTION FROM ADHESION OF THE SMALL COLON. The patient was a bay gelding, which, when received, was suffering from colicky pains.

The manifestation of pain was not violent, but rather persistent, with, at times, intermissions of quietude. The symptoms were such as to indicate impaction of some part of the intestinal tract, and such was the diagnosis. Rectal examination was said not to have given any definite information. During the five days of the animal's treatment he received almost every remedy that can be used in such cases—saline purgatives, eserine, pilocarpin, arecolin, clysters, etc., but only with negative results. A post-mortem revealed an unusual alteration. About eighteen inches of the first convolution of the small colon was doubled on itself, like, for illustration, the loop on one side of a double bow on a shoestring, the two ends of the loop of intestine being united by a short, thick ligament composed of fibrous tissue and peritoneum, and extending from one side to the other. The anterior attachment of this ligament was confounded with the peritoneal fold extending from the ileum to the origin of the small colon. The fourth portion of the folded colon and the origin of the floating colon were distended, but behind the doubled convolution of the small colon the intestine was empty. It seems peculiar that this anomaly should arrest peristalsis and the passage of the food at this point, unless this was due to the dryness of the latter; but no other cause of obstruction could be found, and the doubling up of that part of the colon evidently occluded its lumen and prevented the passage of the intestinal contents.

---

The Veterinary Department of the Minnesota State Board of Health, under Dr. Reynolds, has again been recognized by the Legislature in a substantial way. The last Legislature increased the previous annual appropriation by $3000. The present Legislature has added $2500, the full amount asked for in each case. The laboratory appropriation will free $1200 more for this department, a gradual increase of $3700 since Dr. Reynolds took up this work in the spring of 1897. An appropriation was also made of $7500 for the support of the State Board of Health bacteriological laboratory. This laboratory is finely equipped and doing good work, but has heretofore been supported out of the other State Board of Health funds, having none of its own.

